# Cardiac Coherence Training to Reduce Anxiety in Remitted Schizophrenia, a Pilot Study

**DOI:** 10.1007/s10484-015-9312-y

**Published:** 2015-09-07

**Authors:** M. Trousselard, F. Canini, D. Claverie, C. Cungi, B. Putois, N. Franck

**Affiliations:** Département des Facteurs Humains, Institut de Recherches Biomédicales des Armées, 24, Avenue des maquis du Grésivaudan, BP 87, 38702 La Tronche Cedex, France; Institut Francophone de FORmation et de Recherche en THErapie Comportementales et Cognitives, 10 avenue Gantin, 74150 Rumilly, France; Fondation Formation universitaire à distance, Suisse, FS-CH, Überlandstrasse 12, 3900 Brigue, Switzerland; Service Universitaire de réhabilitation, Université de Lyon (Université Lyon 1), Centre Hospitalier Le Vinatier, 98 rue Henri Boileau, 69006 Lyon, France

**Keywords:** Schizophrenia, Anxiety, Well-being, Emotional regulation, No drug therapy

## Abstract

Health care that addresses the emotional regulation capacity of patients with schizophrenia confronted with daily stress may contribute to a less anxious life. A psycho-physiological training [cardiac coherence training (CCT)] focusing on emotion regulation is known to decrease anxiety for healthy individuals. We performed a pilot cross sectional survey to explore the benefits of CCT for clinically stable patients with schizophrenia. Ten patients were enrolled in the program consisting of twelve weekly 1-h session programs monitored over a 2-month period. Standardised questionnaires were used before and after the intervention to assess anxiety, well-being outcomes, and how patients deal with stress and stressors. Results showed that this quite-well accepted intervention improved (or tended to improve) well-being outcomes, state-anxiety, and emotional stressors evaluation. The successful transformations were higher for patients with the highest clinical and emotional suffering. Thus, this pilot study revealed that CCT may help patients with schizophrenia to deal with anxiety in daily life.

## Introduction

Schizophrenia (SCZ) affects cognition, emotions (Aleman and Kahn 2005; Ross et al. 2006; Walker et al. 2004), and social functioning (Walker et al. 1984; Zuroff and Colussy 1986; Hooker and Park 2002). As well as the neurobiological mechanisms involved in schizophrenia, stress is considered to be highly involved in its time course in ecological conditions. Firstly, exposure to environmental and psychosocial stressors may be risk factors in the development of schizophrenia, as proposed in the stress-vulnerability model of schizophrenia (Corcoran et al. 2003; Duncan et al. 1999; Fowles 1992; Nicholson and Neufeld 1992; Nuechterlein and Dawson 1984). Stress during development has been suggested to facilitate schizophrenia (Bennett 2008). Furthermore, exposure to stressful life events seems related to a variety of symptoms (Dohrenwend and Egri 1981; Norman and Malla 1993, 1994; Ventura et al. 1989). Actually, exposure to a stressful family milieu interacts heavily on the time course of schizophrenia (Falloon and McGill 1985; Butzlaff and Hooley 1998). It is therefore not surprising that patients with schizophrenia suffer from high anxiety levels (Lewis and Lieberman 2000). The mechanism by which anxiety occurs and how patients can cope with it remains incompletely understood.

In schizophrenia, emotional disturbances lead to anxious comorbidities (Braga et al. 2004; Lysaker and Salyers 2007; Achim et al. 2011). They severely alter self-esteem (Karatzias et al. 2007; Lysaker et al. 2010), quality of life (Huppert et al. 2001; Braga et al. 2005), cardiovascular parameters (Townsend et al. 2007), and favour associated drug abuse (Goodwin and Amador 2003). They are theorized as a disjunction between expression, perception and experience of emotion rather than a general, unidimensional reduction or increase of emotional processing (Aleman and Kahn 2005; Kring and Neale 1996; Kring and Earnst 1999). Facing an intense emotional experience, the subjects with schizophrenia behave as if their emotional perception and expression are reduced (Aleman and Kahn 2005). On the other hand, exposure to a neutral stimulus produces excessive arousal responses and an autonomic imbalance, suggesting an increase in the emotional experience (Berger et al. 2010). Although the former characterizes emotional blunting, which is a core feature of SCZ, the latter may address an aberrant assignment of salience to otherwise insignificant stimuli. This could be the result of the patient’s inner world felt as threatening or the abnormal codification of the stimulus, its novelty being more important that its value. Furthermore, it is attracted to patients’ poor interpersonal and social functioning (Walker et al. 1984; Zuroff and Colussy 1986; Borod et al. 1993; Hooker and Park 2002) and severely affects the patient’s life.

The problem therefore is to limit the perceived threat of the world using interventions aiming to help patients to cope with negative emotions and stress in daily situations. Stress management programs may focus on cognitive processing in order to improve problem-solving skills (Starkey et al. 1995). They may also focus on psychosocial processing using interpersonal interactions in a social activities program like cooking, cultural visits etc., in order to encourage patients to provide mutual support for one another. Alternatively, it could focus on the interoceptive processes that may reduce perceived stress when facing daily stressful situations.

Several lines of evidence suggest that interoceptive awareness (awareness of the body’s state) is a key element for insight in SCZ (David et al. 1992; Gilleen et al. 2011; Palaniyappan et al. 2011). Indeed, interoceptive information forms the basis of various higher order processes such as self-awareness, mental phenomenon, discrimination between self-generated and external information (Frith et al. 2000; Franck et al. 2001), and time perception (Craig 2010; Wittmann et al. 2010). Although heterogeneous findings suggest a relationship between interoception-related dysfunction and anxiety disorders in SCZ, to our knowledge data focusing on this specific topic are scarce. However, studies have already shown that intervention based on interoception, such as decrease in muscle tone (Chu et al. 2009), mindfulness (Davis et al. 2007), and yoga (Vancampfort et al. 2011), are anxiolytic for schizophrenia.

The purpose of this study was to evaluate the interest of an emotional experience intervention for remitted patients with schizophrenia medicated by minimal effective doses of antipsychotics by using an emotional and interoceptive biofeedback intervention on anxiety feelings. This intervention aimed to bypass cortical cognition to directly offer a congruent corporal sensation that could reduce anxiety faced in stressful situations.

## Materials and Methods

### Subjets

Ten subjects, five women and five men, all volunteers, were included in the study. They were all clients of the rehabilitation center for psychotic disorders (Le Vinatier hospital) situated in Lyon, France. Criteria for entry into the study included: a DSM-IVR diagnosis of schizophrenia as confirmed by the Mini International Neuropsychiatric Interview for DSM-IV (MINI; Lecrubier et al. 1997); aged between 18 and 65; and clinically stable (i.e. not having required hospitalization or increases in medication as a result of an exacerbation of acute symptoms over the previous 3 months. This study was approved by the human ethics committees from the university hospitals at Le Vinatier and Grenoble, France. Identified for ClinicalTrials.gov is NCT02390271. In compliance with the Helsinki Convention, which controls and regulates human experimentation, informed consent was obtained from all subjects.

### Materials

All subjects completed a set of “paper and pencil” standardized assessments at pre-intervention and immediately after the end of the intervention. Although there is evidence that patients with SCZ are as competent as non patients in their ability to subjectively appraise and describe their feelings in a coherent manner (Betensky et al. 2008; Grant et al. 1976; Horan and Blanchard 2003), patients completed the questionnaires with a nurse’s help to correctly understand the items, if necessary.

Trait and State anxiety was assessed using the French version of the Spielberger State-Trait-Anxiety Inventory (STAI-Y). It is a 40-item 4-point (1–4) self-report questionnaire (Spielberger 1975; Bruchon-Schweitzer and Paulhan 1993). In the state portion of the scale, 20 items allow subjects to report the extent of their anxiety at particular moments. In the trait scale, the remaining 20 items allow respondents to indicate the intensity of their anxiety in general. The alpha coefficients for the trait and state scales were .93 and .62, respectively (Spielberger 1983). From French data, the threshold is set for a score higher than 47 for the trait scale and 41 for the state scale (Bruchon-Schweitzer and Paulhan 1993). Both scores were computed in this study.

Three questionnaires were used in order to evaluate the outcomes of the patients’ quality of life.

The Positive And Negative Syndrome Scale (PANSS) was used, first to assess the severity of the illness, as perceived by the clinical staff. The PANSS is a 30-item 7-point (1–7) rating hetero-questionnaire, which amalgamated the 18-item BPRS and 12 items from the Psychopathology Rating Schedule (Guelfi 1997; Kay et al. 1987). The items were precisely defined, as were anchor points for the numerical rating of each item. The PANSS was divided into three subscales evaluating positive, negative and general psychopathology sub-scales. The alpha coefficients for the positive and negative scales were .73 and .83, respectively. The psychopathology scale similarly revealed high internal consistency (alpha coefficient of .79; Kay et al. 1987). Although no consensual cut-off was considered, sub-scale scores were associated with a number of clinical, treatment and cognitive variables, including premorbid adjustment, but not an outcome per se (Mortimer 2007).

Next the patient’s body/mind perceptions were assessed using mindfulness concept. Respondents’ mindfulness levels were assessed using the French version of the short form of the Freiburg Mindfulness Inventory-14 (FMI), which is a 14-item 4-point (1–4) self-report questionnaire developed for people with no background knowledge about mindfulness (Trousselard et al. 2010; Walach et al. 2006). It is a consistent and reliable scale evaluating several important aspects of mindfulness, and is considered as one-dimensional for practical purposes (Kohls et al. 2009; Walach et al. 2006). Validation studies have reported internal consistency (alpha) coefficients ranging from .74 to .79 (Trousselard et al. 2010; Walach et al. 2006). Depending on the suggested time frame, it can be used to assess state- or trait-like components. For the purposes of this study, this short form was used for measuring respondents’ mindfulness state. From the French health population, threshold is set for a state score higher than 37.24 (Trousselard et al. 2010).

Thirdly, patients’ quality of life was evaluated using the Warwick–Edinburgh Mental Well-being Scale (WEMWBS; Tennant et al. 2007), a 14-item 5-point (1–5) self-report questionnaire in which individuals respond to questions about their thoughts and feelings. The French translation validation process is ongoing (Trousselard et al. in revision). Validation studies have reported internal consistency (alpha) coefficients ranging from .85 to .90 for adult samples (Tennant et al. 2007; Trousselard et al. in revision) and of .88 for clinically stable patients with schizophrenia (Trousselard et al. in revision). No cut-off was defined for well-being. This questionnaire was included in the report submitted to Mental Health Research Network (2010) concerning the consensus about the measures of general mental health outcomes which service users judge to be appropriate and relevant for assessing people with psychosis and mood disorder.

One questionnaire was used to assess how patients deal with stress and stressors. The Derogatis Stress Profile (DSP; Derogatis 1987; Montgomery et al. 2002; 15 min) is a 77-item Likert scale self-report questionnaire assessing cognitive and emotional stress. The DSP targets three domains of stress: (1) environmental events, (2) personality mediators, and (3) emotional responses. Each of these domains includes several dimensions. Internal consistency across the stress dimensions used in this analysis as determined using coefficient alpha was .75 for patients and .82 for healthy volunteers (Derogatis 1987). No cut-off was defined for DSP sub-scales. Compared with healthy volunteers significant increases in stress were observed among patients with a first episode of schizophrenia for the domestic environment events appraisal, driven behaviour personality mediators and depressive emotional responses (Betensky et al. 2008). The vocational environment items were excluded from analyses given the potential probability that patients would not be working because they were ill.

### Intervention Program

The cardiac coherence training (CCT) incorporates a series of emotion-refocusing and restructuring techniques developed by the Institute of HeartMath (Boulder Creek, CA). The CCT involves the use of biofeedback to control and visualize heart rate variability (HRV)—the moment-to-moment change in heart rate (McCraty 2001). Cardiac coherence is the coupling and synchronization of the rhythm of breathing to the rhythm of the heart. An efficient HRV is obtained when breathing is at the resonant frequency of the baroreflex system (McCraty 2001). The use of this practice in daily life has been shown to improve health because of the adjusted autonomic system balance (McCraty et al. 1995, 1998). This balanced state affects the condition of all other systems under ANS control: respiratory, cardiac, digestive, immune systems, and produced a general feeling of wellness (Barrios et al. 1997; Luskin et al. 2002).

The general training includes in an individual adjustment using the following techniques: biofeedback-assisted breathing classes that erase negative emotion involving the cardiac and limbic neural pathways, controlling one’s own and other’s emotions, mastering positive cognition and enhancing self-conception, anchoring positive memories, learning how to recognize psychological reversal in others. For SCZ training, the exercises were simpler and easier. First, patients received exercises to make heart rate fluctuation more rhythmic or coherent with the help of the clinician trained in CCT. This step helped to put Heart Focused breathing^®^ and Neutral Quick coherence^®^ exercises from the HearthMath program into practice. Secondly, the patients learned to appreciate themselves, to substitute stressful responses with more positive emotions, and to more freely engage in the caring side of others using Freeze-Frame^®^ and Heart locking^®^ training. At each step, the cardio biofeedback was used for strengthening patients. The schedule was gradually advanced as part of a personalised program based on an 8–12 week 1-h session for complete training. There were also daily homework relaxation exercises to further deepen emotion regulation during ordinary daily activities. No assessment of daily practice was recorded, but each patient was questioned on the frequency of his/her daily practice outside with sympathetic understanding. Assessments were realised the week before the first training session and the week following the last training session.

### Statistical Analysis

All data, expressed as mean (SD), were treated as ordinal data except for the sex, tobacco use, and number of hospitalizations. A principal components analysis (PCA) was first performed to detect any redundancy between psychological outcome variables (i.e., PANSS, STAI trait and state, mindfulness, and well-being) and to reduce them into a smaller number of artificial principal components (Abdi and Williams 2010). The results support a robust single principal component explaining 72.33 % of the variance. This artificial variable indicated that subjects with high scores in PANSS, STAI, also exhibited low scores in mindfulness and well-being. Such subjects presented positive outcomes according to this new artificial variable whereas the remaining had negative outcomes. PCA results were then used to categorize subjects in negative or positive outcomes using the K-means clustering method. Then, the two groups were defined as a group with negative outcomes (GNO) and a group with positive outcomes (GPO; Table 1).

**Table 1:** Respondents’ characteristics [mean (SD)] for socio-demographic and psychological outcomes’ variables for the schizophrenia sample, and negative and positive outcomes’ groups (see Sect. 2 for more precisions)

	Full sample (n = 10)	GNO (n = 5)	GPO (n = 5)	Χ^2^ values	*p*
Sex (men/women)	5/5	3/2	2/3	.36	.548
Age (years)	34.86(10.89)	29.75(6.8)	41.66(12.85)	2	.157
Duration of SCZ	6.11(6.27)	9.2(7.15)	2.25(.95)	3.43	.066
Number of hospitalizations	2.37(1.06)	2.75(.5)	2(1.41)	1.09	.294
Family help (yes/no)^a^	8/1	3/1	5/0	1.25	.263
Tobacco use (yes/no)^a^	4/9	2/2	2/3	.07	.794
PANSS	74.9(19.74)	85.2(15.16)	64.6(19.55)	2.47	.106
Positive symptoms^b^	15.8(6.71)	16.2(7.19)	15.4(7.02)	.44	.833
Negative symptoms^b^	22.2(9.07)	27.4(5.12)	17(9.56)	2.46	.106
General Psychopathology^b^	36.9(8.43)	41.6(6.81)	32.2(7.66)	2.15	.142
STAI-trait	47.8(14.11)	59.2(10.2)	36.4(4.33)	6.9 p	.008
STAI-state	46.2(16.07)	59.8(8.46)	32.6(6.88)	6.82	.009
Mindfulness	34(9.79)	27.6(9.93)	40.4(3.84)	4.06	*.043*
Well-being	44.4(14.58)	32.6(7.63)	56.2(8.47)	6.82	*.009*
Personality mediators	46.12(5.62)	47.75(4.85)	44.5(6.56)	.35	.554
Emotional responses	53.62(10.48)	64.5(10.66)	42.75(11.41)	3.06	.081
Anxiety	49.5(15.99)	59.25(16.37)	39.75(8.65)	2.55	.101
Hostility	50.62(12.61)	57.25(7.18)	44(14.23)	1.73	.188
Depression	59.62(12.98)	69.25(8.06)	50(9.02)	5.39	*.020*

*p* values indicated in italic for significant (or tendency) differences

^a^There were missing data for one subject

^b^The subscales of the PANSS were not taken into account in the clustering analysis

The second step of the statistical analysis was carried out using a non-parametric test, due to the small size of the groups, for assessing the differences between groups on socio-demographic as the stress and stressors variables.

Lastly, the effect of the CCT was performed using analyses of variance (ANOVA) with time session (before and after CCT) as within-subjects effect, and group (GNO and GPO) as between-subjects effect. This was done separately for each psychological variable. For significant interaction between within- and between-subjects, post hoc analyses using Newman–Keuls were applied. All analyses were performed with SPSS 17.0 for Windows (SPSS GmbH Software, Munich). We judged *p* < .05 as significant. When *p* ≤ .1, results expressed a tendency to a difference.

## Results

### Socio-demographic Sample (Table 1)

The descriptive findings for the full sample of 10 respondents (five men and five women; mean age was 36.25 (9.57) years showed that mean duration of illness was 6.1 (6.27) years and mean number of hospitalization was 2.37 (1.06). Four subjects were smokers. All patients were under antipsychotic treatment (risperidone, olanzapine, clozapine, haloperidol or levomepromazine). The two groups, as defined by the cluster classification, were demographically similar in terms of the assessed socio-demographic data. Duration of illness failed to be different between groups (*p* = .07).

### Psychological Scores According to the Groups

As shown in Table 1, the GNO exhibited higher scores for the trait-anxiety, and state-anxiety scales associated with lower scores for the mindfulness and well-being scores than the GPO.

Differences failed to be significant for the general PANSS score (*p* = .1), for the negative (*p* = .11) symptoms PANSS scores, and for the stress and emotional stressors DSP score (*p* = .08). Lastly, no difference was observed for the environmental perception as the personality mediator stressors.

### CCT Acceptation According to the Group

Whatever the group, all subjects wanted to practice during the time of the training. As they should not be obliged to, the regular presence indicated that the CCT was accepted by patients. Moreover, more than half of the sample did not want to stop the training at the end of the experience.

### Evolution of Outcomes, and the Stress and Stressors Scores After CCT: Intervention Efficiency

**Fig. 1:**
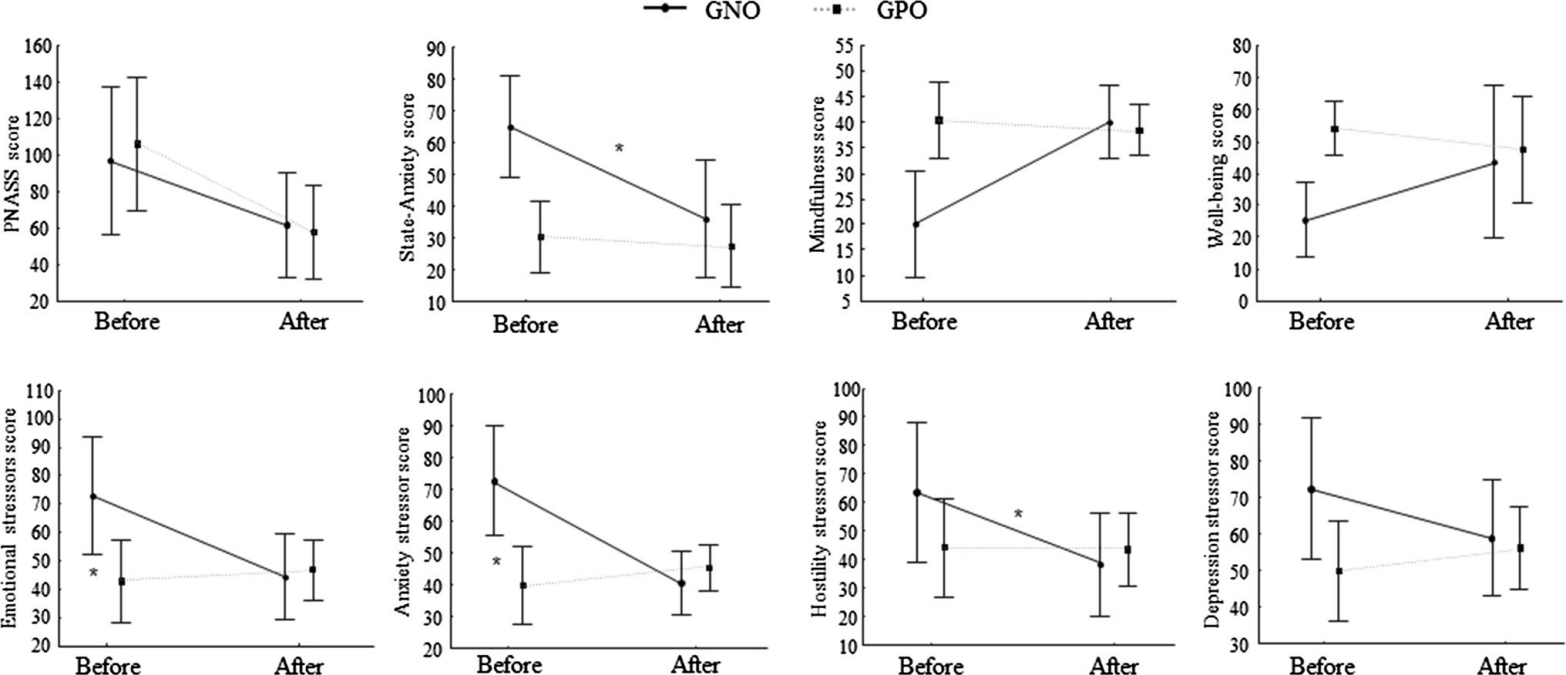
Evolution of the assessed outcomes’ scores for the GNO and GPO after the CCT. *indicating a significant difference (*p* < .05)

Successful transformation into outcomes (Fig. 1) was observed for the mindfulness [*F*(1) = 8.38, *p* = .04] score after the CCT. Differences failed to be significant for the PANSS [*F*(1) = 5.28, *p* = .08], and for anxiety-state [*F*(1) = 5.48, *p* = .08]. No effect was observed for the well-being score [*F*(1) = .93, *p* = .38]. Interestingly, the interactions’ results highlighted an improvement in mindfulness scores only for the NGO [*F*(1,4) = 14.81, *p* = .01]. Interactions failed to be significant between group and anxiety-state [*F*(1,4) = 5.428, *p* = .08], as between group and well-being [F(1,4) = 3.5, *p* = .13].

With regard to stress and stressors effects of the CCT (Fig. 1), results showed no effect for environmental as the personality-mediators factors but a significant improvement for the emotional stressors [*F*(1) = 6.66, *p* = .05], as for anxiety-emotional stressor [*F*(1) = 9.65, *p* = .03]. For hostility-emotional stressors, the difference failed to be significant [*F*(1) = 5.75, *p* = .07]. Lastly, no effect was observed for the depression score [*F*(1) = .26, *p* = .63].

Interestingly, the interactions’ results highlighted an improvement in emotional stressors score only for the NGO [*F*(1,4) = 7.81, *p* = .04]. More precisely, for emotional sub-factors, a significant interaction was found for the anxiety emotional variable [*F*(1,4) = 7.81, *p* = .04] with an higher decrease in anxiety level for the GNO group. Decrease in hostility-emotional score for the GNO failed to reach a significance [*F*(1,4) = 5.3, *p* = .08]. Moreover, no effect was observed for the depression-emotional subscale [*F*(1,4) = 1.97, *p* = .23].

## Discussion

The purpose of this study was to evaluate the effect of the interoceptive awareness intervention using CCT on anxiety feelings for patients with remitted schizophrenia. Results showed that the intervention induced encouraging transformation into emotional and interoceptive outcomes that imply a better quality of life of patients with SCZ. Although no significant improvement was observed for the PANSS scores, improvement in the mindfulness score was observed. It can be noted that this, non-invasive treatment was accepted by patients since none of them discontinued the training.

By taking into account how the interoceptive therapeutic approach deals with stress and stressors, the second results showed that the emotional stressors perception was improved whereas no effect was observed concerning personality-mediator stressors as environmental stressors perceptions, as assessed by the DSP. The DSP paradigm treats stress as a process and enables the clinician to evaluate how patients subjectively appraise the influence of real-life stressors. More precisely, environmental stressors tested the need to act constructively, namely in interpersonal appraisal (Betensky et al. 2008), and the personality-mediators were associated with a vulnerability personality trait (Betensky et al. 2008; Horan et al. 2005; Nuechterlein et al. 1992). Taken together, these data may indicate that such an interoceptive therapeutic approach may act on the emotional feelings without changing personality as cognition. This was in accordance with the hypothesis that intervention may act to bypass cortical cognition to directly offer a congruent corporal sensation that could reduce the perceived stress facing stressful situations.

Furthermore, data highlighted that clinically stable SCZ must not be considered as a homogenous sample. Whereas the clinical scale alone failed to separate patients at baseline, an operative discrimination between two groups of patients appears by taking into account clinical as emotional and interoceptive assessments. Indeed, the cluster analyses applied to this set of questionnaires led to categorizing subjects in negative or positive outcomes. The group with negative outcomes exhibited lower mindfulness, and well-being scores and higher state-anxiety score at baseline. The GNO also tended to exhibit higher emotional stressors at baseline on the DSP scale, namely a significantly higher level of depression. Furthermore, the two groups did not differ on the environmental perception level, as on the personality mediator stressors level. However, they exhibited a higher score in trait-anxiety. According to the literature, this negative outcomes’ group may be considered to be at a higher risk for relapse according to the usual key role given to the stress in the pathophysiology of SCZ (Kanner et al. 1981; DeLongis et al. 1982; Norman and Malla 1994). Consequently, the higher anxiety emotional reduction, and better body/mind feeling for NGO appears as a very important benefit for these patients characterized by a high risk of pejorative evolution. The fact that such an improvement was not observed for the positive outcomes group may be considered as normal as their scores for the outcomes’ variables were in the normal range (Bruchon-Schweitzer and Paulhan 1993, Tennant et al. 2007; Trousselard et al. 2010).

There were several limitations to this study that preclude firm conclusions. One first limitation is due to the small sample size. Given the number of statistical comparisons, the efficiency of CCT should be accepted with caution. Results, secondly, need further investigations, namely randomized controlled studies. The third limitation concerns the absence of control of antipsychotic drugs duration, as patients with a short duration of antipsychotic drugs exposure were found to have a significantly higher hostility level than patients with longer exposure (Betensky et al. 2008). Furthermore, the duration of the positive effects on the outcomes’ improvement were not assessed. Finally, mechanisms were not evaluated in this study, thus correlations between emotional processing, cognitive dysfunctions, clinical characteristics, and brain functioning before and after CCT need to be further investigated.

To summarize, our findings show that, the interoceptive intervention using CCT with biofeedback is accepted by clinically stable schizophrenic individuals. The CCT induced no delusion or other psychotic symptoms, even for patients with the highest negative outcomes, as anxiety. Rather, this mind/body therapeutic approach appears to help patients to deal with emotional experience and improves some assessed outcomes. Moreover, the efficiency may appear to be more efficient than the patients being at risk of relapse. Taking into account that the intervention may help patients have a better quality of life, the observed improvements have been attributed to an improvement in the emotional functioning whereas cognitive appraisals were unchanged. By increasing self-awareness levels, it involves teaching individuals’ skills that improve patients’ ability to attend to their experience in the present moment while suspending judgment and to purposefully shift their attention, the use of the interoceptive therapeutic intervention has led to important emotional behavior change. CCT, even if it needs to be further investigated, could be considered as a potentially effective and safe approach to care for patients who are disabled by schizophrenia and suffer from their emotional outcomes.
